# Lymphocytic Interstitial Pneumonia Presenting as an Enlarging Pulmonary Nodule in a 65-Year-Old Woman: A Case Report

**DOI:** 10.7759/cureus.88787

**Published:** 2025-07-25

**Authors:** Nguyen Cuong Pham, Nhu Huy Pham, Duc Phu Tran, Minh Tri Ngo, Nguyen Thanh Xuan, Cong Thuan Dang, Nguyen Huu Son

**Affiliations:** 1 Pathology, Hue Central Hospital, Hue, VNM; 2 Osteopathic Medicine, Arizona College of Osteopathic Medicine, Midwestern University, Glendale, USA; 3 Nephrology and Dialysis, Thong Nhat Hospital, Ho Chi Minh, VNM; 4 Radiology, Hue Central Hospital, Hue, VNM; 5 Abdominal Emergency and Pediatric Surgery, Hue Central Hospital, Hue, VNM; 6 Pathology, Hue University of Medicine and Pharmacy, Hue, VNM; 7 Pediatrics, Hue Central Hospital, Hue, VNM

**Keywords:** benign lymphoproliferative disorder, immunohistochemistry, lung, lymphocytic interstitial pneumonia, pulmonary nodules

## Abstract

Lymphocytic interstitial pneumonia (LIP) is a rare, benign lymphoid interstitial lung disease that frequently masquerades as malignancy on imaging and even frozen‑section histology. We report the case of a 65‑year‑old Vietnamese woman who presented with chronic right‑sided chest pain and a nonproductive cough. Baseline high‑resolution computed tomography (HRCT) revealed a 28 × 20 mm irregular opacity in the right upper lobe with scattered bilateral micronodules, all of which proved refractory to empirical antibiotics. Ten months later, the dominant lesion had enlarged to 36 mm, prompting video‑assisted right upper lobectomy for definitive diagnosis. Histopathology demonstrated diffuse lymphoplasmacytic infiltration of the alveolar septa with prominent lymphoid follicles. Immunohistochemistry showed CK7‑ and TTF‑1‑positive epithelial elements surrounded by CD20‑positive B‑cell follicles and scattered CD3‑positive T‑cells, with a Ki‑67 index < 5%. Although light chain restriction analysis was not performed, the histologic and immunophenotypic features supported a diagnosis of LIP. The patient recovered uneventfully and, at 12‑month follow‑up, remains asymptomatic without radiologic progression. This case underscores the importance of integrating advanced imaging with comprehensive immunopathology to distinguish LIP from carcinoma or lymphoma, thereby preventing unnecessary aggressive treatment, and it adds to the limited Asian literature on this uncommon entity.

## Introduction

Lymphocytic interstitial pneumonia (LIP) is an uncommon benign lymphoproliferative disease first described by Carrington and Liebow in 1966 [1]. It is characterized histologically by diffuse infiltration of alveolar septa by polyclonal lymphocytes, plasma cells, and histiocytes, often with lymphoid follicle formation. Clinically, LIP occurs sporadically or in association with autoimmune conditions such as Sjögren’s syndrome and immunodeficiency states, including human immunodeficiency virus (HIV) infection [2,3].

Despite its benign nature, LIP poses a diagnostic challenge due to its nonspecific clinical presentation and overlapping radiologic features with malignant lymphoma, lymphangioleiomyomatosis, and other interstitial or cystic lung diseases [4,5]. This frequently leads to misdiagnosis as malignancy, particularly when thin‑walled cysts or nodular infiltrates are present. Because routine imaging and even frozen‑section histology may suggest a neoplastic process, definitive diagnosis requires thorough immunopathologic evaluation. We report the first histologically confirmed case of LIP in Vietnam and provide a concise review of the current literature to highlight diagnostic pitfalls and management considerations.

## Case presentation

Clinical history and examination

In May 2023, a 65‑year‑old female patient presented with intermittent right‑sided chest pain and a mild nonproductive cough. She was a lifelong nonsmoker and denied systemic symptoms. Physical examination was unremarkable; Eastern Cooperative Oncology Group performance status was 1.

Laboratory investigations

Laboratory results were unremarkable (Table 1). Complete blood count, serum biochemistry, and autoimmune profile (antinuclear antibody (ANA), anti-Sjögren's syndrome A (anti‑SSA), anti-Sjögren's syndrome B (anti‑SSB)) were within normal limits. HIV serology and interferon‑γ release assay for tuberculosis were negative.

**Table 1:** Laboratory findings at presentation anti-SSA: anti-Sjögren's syndrome A; anti‑SSB: anti-Sjögren's syndrome B; HIV: human immunodeficiency virus

Parameter	Patient value	Reference range
White blood cell count (×10^9^/L)	6.8	4.0-10.0
Hemoglobin (g/dL)	13.2	12.0-16.0
Platelet count (×10^9^/L)	235	150-400
C‑reactive protein (mg/L)	3	
Antinuclear antibody (ANA)	Negative	Negative
Anti‑SSA	Negative	Negative
Anti‑SSB	Negative	Negative
HIV serology	Negative	Negative
Interferon‑γ release assay	Negative	Negative

Imaging findings

**Figure 1:**
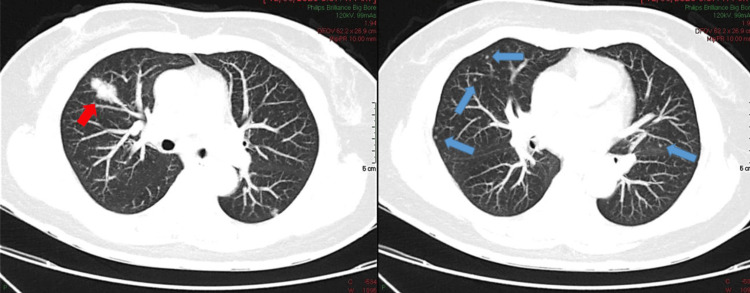
Baseline axial HRCT showing a 28 × 20 mm irregular opacity in the right upper lobe (red arrow) with multiple bilateral micronodules (blue arrows) HRCT: high‑resolution computed tomography

High‑resolution computed tomography (HRCT) revealed a 28 mm spiculated mass in the anterior segment of the right upper lobe, accompanied by diffuse micronodules (Figure 1). No pleural effusion or mediastinal lymphadenopathy was detected.

Initial management and follow‑up

**Figure 2:**
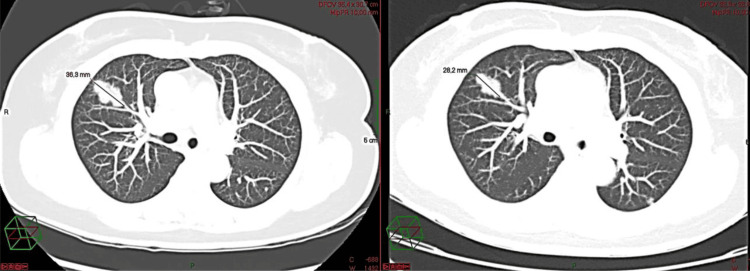
Follow-up HRCT 10 months later revealing interval growth of the right upper lobe lesion to 36 mm

The case was reviewed at a multidisciplinary tumor board, which recommended surgical resection due to lesion progression and nondiagnostic imaging features. Empirical levofloxacin and amoxicillin-clavulanate were prescribed for presumed chronic pneumonia. A follow‑up HRCT in March 2024 demonstrated enlargement of the lesion to 36 mm with persistent micronodules (Figure 2). Owing to technical constraints, percutaneous and bronchoscopic biopsies were not feasible; thus, video‑assisted thoracoscopic right upper lobectomy was performed for definitive diagnosis.

Pathology

**Figure 3:**
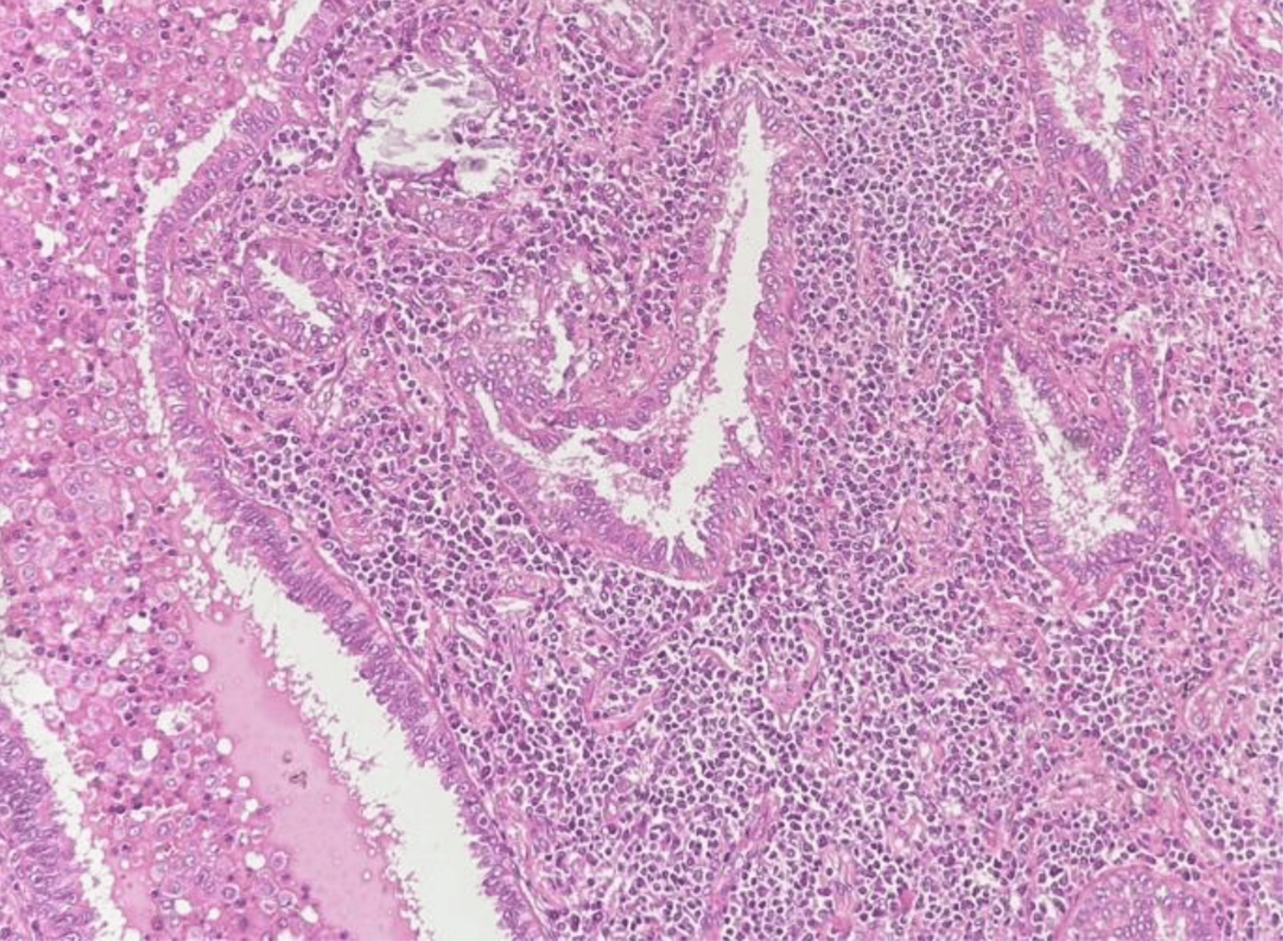
Representative histopathology of excised lung tissue (H&E, ×200) showing diffuse lymphoplasmacytic infiltration of alveolar septa and interstitial tissue with lymphoid follicle formation, consistent with lymphocytic interstitial pneumonia (LIP) H&E: hematoxylin and eosin Immunohistochemical findings (not shown) demonstrated CK7, TTF-1, CD20, CD3, p63, and low Ki-67 expression supporting the diagnosis

Macroscopically, a firm tan-white mass measuring 3.6 cm in greatest dimension was noted. There was diffuse infiltration of the alveolar septa, interlobular interstitium, and peribronchiolar regions by small lymphocytes, plasma cells, and histiocytes, with scattered reactive lymphoid follicles. No epithelioid granulomas were observed. Atypical epithelial inclusions raised concern for lymphoepithelial carcinoma. Immunohistochemistry demonstrated CK7- and TTF-1-positive epithelial cells, CD20-positive B-cell follicles, scattered CD3-positive T-cells, p63-positive basal cells, and a low Ki-67 index (<5%), supporting a diagnosis of LIP. Although kappa/lambda light chain restriction and Epstein-Barr virus-encoded RNA (EBER) were not performed, the combined morphologic and immunophenotypic findings were characteristic of LIP. Due to technical constraints, only representative H&E histology is shown in Figure 3.

Outcome and follow‑up

The postoperative course was uneventful. At 12 months, the patient remains asymptomatic; HRCT shows no recurrence or new lesions. She continues to follow up every three months.

## Discussion

LIP remains diagnostically challenging due to its rarity and variable radiologic and histologic presentations. In particular, LIP can mimic other cystic lung diseases, such as Birt‑Hogg‑Dubé syndrome and lymphangioleiomyomatosis. A recently proposed algorithmic approach recommends distinguishing these entities based on features such as cyst distribution, associated nodules, and clinical context (e.g., family history, cutaneous lesions, or tuberous sclerosis) [6]. Epidemiologically, the prevalence of LIP is estimated at fewer than two cases per million person‑years, with a marked female predominance and peak incidence between the fifth and sixth decades of life. The largest clinicopathological cohort to date, comprising 126 cases, confirmed this demographic distribution [7].

Pathogenesis and histopathology

Current evidence supports an antigen‑driven, polyclonal lymphoid reaction expanding the interstitium and peribronchiolar regions. The 2021 WHO classification of thoracic tumors recognizes LIP as a distinct benign lymphoproliferative process [8]. Clonality studies in the literature consistently demonstrate mixed kappa- and lambda-light-chain plasma cells, and EBER may be detectable in scattered B-cells. In our case, EBER in situ hybridization and light chain restriction analyses were not performed; thus, the diagnosis was based on morphology and immunophenotype alone. These features, along with low proliferative activity, help differentiate LIP from pulmonary mucosa‑associated lymphoid tissue (MALT) lymphoma. In our case, the low Ki‑67 index and preservation of the epithelial basement membrane on cytokeratin staining were persuasive arguments against neoplasia.

Radiologic-pathologic correlation

HRCT typically shows a triad of ground‑glass opacities, thin‑walled cysts, and ill‑defined centrilobular nodules. When LIP is associated with Sjögren’s syndrome, cysts are usually basal and peripheral, whereas nodules predominate in the mid‑zones [9]. Serial imaging demonstrates that discrete nodules can evolve into cysts over months to years, paralleling lymphoid infiltration and bronchiolar remodelling.

In our case, the radiologic features, particularly the gradual enlargement of a spiculated right upper lobe lesion and the presence of diffuse bilateral micronodules, correlated closely with the histologic findings of dense lymphoplasmacytic infiltration and well-formed lymphoid follicles. The absence of lymphadenopathy or cavitation further supported a benign lymphoproliferative process rather than malignancy. This radiologic-pathologic congruence reinforces the diagnostic value of integrating imaging with histopathology in distinguishing LIP from mimics such as lymphoma or organizing pneumonia.

Differential diagnosis

The principal mimickers are pulmonary MALT lymphoma, lymphangioleiomyomatosis, and Langerhans‑cell histiocytosis. Clues favoring LIP include a symmetric basal distribution, widespread cystic change, and polyclonal lymphoid infiltrates with preserved architecture. Large consolidations, bronchovascular bundle thickening, and monoclonality support lymphomas.

Management strategies

There are no randomized trials guiding therapy. Historical series report a 40-70% radiologic response to systemic corticosteroids [10]. Cyclophosphamide has yielded partial responses in steroid‑refractory disease [11], whereas small case series and individual reports suggest that rituximab may induce durable remission in patients with severe hypoxaemia or common variable immunodeficiency [12]. Observation alone is appropriate for asymptomatic or incidentally detected cases, particularly when an underlying trigger can be treated directly. Surgical intervention is generally reserved for diagnostic clarification or focal complications.

Prognosis and follow‑up

Ten‑year overall survival exceeds 80% in idiopathic LIP [13]. However, a 5-7% risk of transformation to lymphoma has been documented in Japanese series [14]. Predictive factors include persistent unilateral consolidation, marked plasmacytosis, and clonal immunoglobulin‑gene rearrangements. We therefore advocate clinical review and HRCT every 3-6 months for the first two years and then annually. Smoking cessation is advisable given the recognized interplay between smoking‑related interstitial lung diseases and lymphoid proliferations [15]. Vigilance is also warranted in people living with HIV, in whom chronic immune activation and opportunistic infections may accelerate progression [16].

Global and regional implications

Reports of LIP from Southeast Asia remain scarce. Our case adds to the limited data and underscores the value of thoracoscopic approaches, including diagnostic lobectomy when biopsy is not feasible, in resource-limited settings. The wider availability of HRCT and immunohistochemistry in Vietnamese tertiary centres should facilitate earlier recognition of LIP and reduce misclassification as tuberculosis or lung cancer.

## Conclusions

This case highlights the importance of considering LIP in the differential diagnosis of enlarging pulmonary nodules with lymphoid histology. Comprehensive evaluation integrating imaging, histopathology, and immunophenotyping is essential to avoid misclassification as malignancy. Where available, advanced bronchoscopic techniques such as transbronchial cryobiopsy or radial endobronchial ultrasound may facilitate earlier, less invasive diagnosis, potentially avoiding the need for surgical resection. Although the prognosis is favorable, vigilant follow-up is advised to detect rare malignant transformation.
